# A Social Network Analysis of Treatment Discoveries in Cancer

**DOI:** 10.1371/journal.pone.0018060

**Published:** 2011-03-28

**Authors:** Athanasios Tsalatsanis, Laura Barnes, Iztok Hozo, John Skvoretz, Benjamin Djulbegovic

**Affiliations:** 1 Center for Evidence-Based Medicine and Health Outcomes Research, University of South Florida, Tampa, Florida, United States of America; 2 Department of Mathematics, Indiana University Northwest, Gary, Indiana, United States of America; 3 Department of Sociology, University of South Florida, Tampa, Florida, United States of America; 4 H. Lee Moffitt Cancer Center & Research Institute, Tampa, Florida, United States of America; University of Zaragoza, Spain

## Abstract

Controlled clinical trials are widely considered to be the vehicle to treatment discovery in cancer that leads to significant improvements in health outcomes including an increase in life expectancy. We have previously shown that the pattern of therapeutic discovery in randomized controlled trials (RCTs) can be described by a power law distribution. However, the mechanism generating this pattern is unknown. Here, we propose an explanation in terms of the social relations between researchers in RCTs. We use social network analysis to study the impact of interactions between RCTs on treatment success. Our dataset consists of 280 phase III RCTs conducted by the NCI from 1955 to 2006. The RCT networks are formed through trial interactions formed i) at random, ii) based on common characteristics, or iii) based on treatment success. We analyze treatment success in terms of survival hazard ratio as a function of the network structures. Our results show that the discovery process displays power law if there are preferential interactions between trials that may stem from researchers' tendency to interact selectively with established and successful peers. Furthermore, the RCT networks are “small worlds”: trials are connected through a small number of ties, yet there is much clustering among subsets of trials. We also find that treatment success (improved survival) is proportional to the network centrality measures of closeness and betweenness. Negative correlation exists between survival and the extent to which trials operate within a limited scope of information. Finally, the trials testing curative treatments in solid tumors showed the highest centrality and the most influential group was the ECOG. We conclude that the chances of discovering life-saving treatments are directly related to the richness of social interactions between researchers inherent in a preferential interaction model.

## Introduction

Randomized controlled clinical trials (RCT) are widely considered one of the most important vehicles of discovery of new treatments. RCTs have been credited with considerable improvement in health outcomes resulting in a significant increase in life expectancy for conditions such as cancer, which is the topic of this paper [1]–[7].

**Figure 1:**
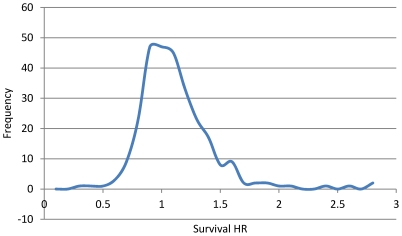
Distribution of treatment success in oncology. Distribution of treatment success in oncology expressed as survival hazard ratio (HR), where higher HR indicates more successful treatments. The curve illustrates slightly increased number of successful treatment consisted with a power law function.

We have previously shown that the success of new treatments in cancer does not fit the random normal distribution curve [8]. We found that new treatments were, on average, slightly superior to standard treatments, bringing about small or moderate advances, with occasional discovery of breakthrough interventions; a pattern of therapeutic discovery that fits a power law distribution (figure 1) [8]. In general, power law distributions describe many natural and man-made phenomena such as the population of cities, the word frequency in a manuscript, the citations of a scientific paper, etc. [9], [10]. The significance of the power law finding in therapeutic discovery arises from the scale free property of the distribution, which implies that, regardless of the number of controlled trials performed, the discovery of new treatments is described by the same power law.

While the power law appears to provide a credible mathematical description of the overall pattern of treatment success, it is not clear what exact mechanism can explain how power law actually works. We have previously argued that trials operate on the borderline of success and failure due to the principle of equipoise [11],which implies that discovery remains possible only if RCTs are performed when there is substantial uncertainty with respect to the relative merits of interventions to be tested. However, if that were the only explanation, the distribution of treatment successes would be random i.e. the pattern of therapeutic discovery would fit the normal distribution, which we found it was not the case. In reality, it could be expected that based on the tremendous amount of effort and money spent on discovery of new treatments, the number of successful RCTs would be significantly greater than the number of unsuccessful ones, resulting in a skewed distribution. The equipoise hypothesis does not provide explanation for the fact that new treatments are slightly more superior to the old ones, as it does not take into account researchers' efforts to develop new more successful treatments [8]. In this paper, we argue that the mechanism responsible for the observed pattern in therapeutic discovery is the social interactions between the researchers who conduct clinical trials (but who do have to work under the ethical requirement of equipoise).

The process of discovery that characterizes scientific progress is inherently a social enterprise. The pursuit of future discoveries depends upon understanding of the existing and ongoing research [12]–[14]. This characteristic of the scientific discovery process has been most memorably captured in the metaphor expressed by Isaac Newton: “*If I have seen a little further it is by standing on the shoulders of Giants*” [15]. Therefore, the process of scientific discovery depends on the interactions between past and current researchers, as well as institutions and the wider scientific community that sanctions the results of a given research endeavor and ultimately ensures that it is accepted [12]–[14]. The same process of social interactions applies to clinical trials, particularly well-designed RCTs.

A trial design is largely attributed to knowledge and information acquired in earlier trials. Investigators tend to interact with colleagues in their immediate environment [16] and/or make use of scientific journals and meetings [17], [18] to share knowledge as well as trial successes and failures among the members of scientific community. However, if testing of new (therapeutic) ideas is to occur, the researchers' personal representations must be further formalized. For example, in the U.S., most RCTs that are not conducted by industry are performed under the auspices of the National Cancer Institute (NCI) that support the co-operative trial infrastructure. All proposals aimed at testing new promising treatments are vetted and ultimately funded through the framework of the NCI co-operative groups (COGs).

We postulate that the social interactions between members of the NCI COGs drive the development of therapeutic discovery for malignant diseases. If this is the case, then the analysis of explicit interactions between RCTs ought to shed some additional light on the treatment discovery process in cancer, in particular, explain the power law pattern of treatment success. Studying the RCTs in such a way is expected to help understand the process of treatment discovery within the entire RCT system that ultimately may help improve health outcomes.

## Methods

### Dataset

We used a data set reported in detail elsewhere [8]. This data set involves 216,451 patients and consists of 624 phase III RCTs sponsored by the NCI COGs conducted and published from 1955 to 2006 [8]. We limit our analysis to 280 out of the 624 trials that considered survival as their primary outcome. In these trials researchers explicitly set out to improve survival by testing new therapeutic agents. These trials used superiority design aiming to address the question if one treatment is superior to another. There were no non-inferiority trials in which success would have been deemed as one treatment being equal or non-inferior to another.

### Treatment discovery

In general, treatment success in cancer can be measured by [8]: (1) assessing the proportion of statistically significant trials favoring new or standard treatments, (2) determining the proportion of trials in which new treatments are considered superior to standard treatments based on investigators' overall judgments, and (3) quantitatively synthesizing data for main clinical outcomes (overall and event-free survival). Each of these measures has its advantages and disadvantages, but, at least, in life-threatening diseases such as cancer, assessing survival seems to be the key determinant of true success rate. Hence, we consider that the best metric of research efforts to discover new, effective treatments is improvement in patients' outcomes. In this work, we choose the survival hazard ratio (HR), as reported in each RCT, as the crucial metric of treatment success. That is, successful trials are considered as those with a statistically significant survival hazard ratio with value greater than 1 (at p value ≤0.05).

### Social Networks

As per our hypothesis, there exists a relationship between RCT interactions and the treatment discovery process. If that is the case, then trials with extensive interactions are expected to be associated with improvements in survival. We used social network analysis to study the effects of these socials interactions on treatment success. An RCT network is represented as a set of nodes, each node denoting a trial, and a set of ties, each tie denoting an interaction between trials. Since it is impossible to determine precisely how RCTs communicate, we assume that RCT interactions could be formed in three ways: (1) based on shared characteristics between trials, (2) based on the treatment success of trials, and (3) at random. We then analyze how treatment success is related to its connections in each type of network.

#### Model 1: RCT interactions based on shared characteristics

**Figure 2:**
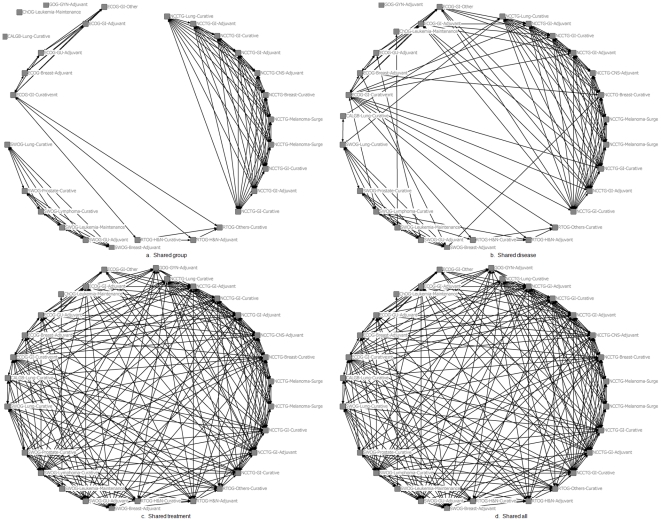
Network configurations of social interactions of RCTs in cancer*. Each node in the network represents a trial cited as the triplet denoting the COG it belongs to, the type of disease and treatment it studies. The networks have been constructed considering relationships between COG (a), type of disease (b), type of treatment (c), and the conjunction of all possible interactions (d). *For illustration reasons, only a limited number of RCTs is shown.

The first model postulates that RCT interactions are confined between trials in related fields. Therefore, interactions between trials occur (a) at the level of each COG (which ultimately proposes the trial to be carried out among the member institutions) (figure 2a), (b) type of disease, since the treatment discovery is typically a disease-oriented process (i.e. breast cancer, gastrointestinal cancer, gynecologic cancer, etc.) (figure 2b), and (c) type of treatment, which defines the category of therapeutic agents (i.e. adjuvant, curative/definitive, induction, etc.) (figure 2c). Ultimately, there are many levels of trial interactions such as the investigator's institution, the study section, the funding source, etc. However, all these types of interactions eventually filter to interactions at the level of COG, type of disease and treatment, which we believe represent the most salient aspects of the RCT system.

The combination of all possible interactions generates 7 different networks, referred to as shared characteristics networks in the rest of the manuscript. In our analysis we have omitted the 3 networks created using interactions at the level of single characteristics since these networks are comprised by isolated groups of trials corresponding to each cooperative group, type of disease, or treatment.

#### Model 2: RCT interactions based on the previous treatment success

The second model theorizes that RCTs interact selectively across the RCT spectrum, specifically, that interactions between the most successful trials are favored (“success breeds success”). Our hypothesis stems from researchers' tendency to interact mostly with established and well known peers. A model for network formation based on such interactions is the preferential attachment model [19], [20]. According to this model, nodes are connected at random with a bias towards the most connected nodes. In our setting, we argue that the most connected nodes are represented by the most successful trials, particularly those with *HR*>1 and *p value*<0.05. Therefore, we construct the RCT network assuming that the probability of an RCT receiving a tie is proportional to the success of the RCT as measured in terms of survival hazard ratio (HR), and the statistical significance of the reported results as indicated by the p value. We call this network the preferential attachment network in the rest of the manuscript.

The preferential attachment RCT network is formed iteratively, starting with a small number of RCTs. At each iteration, a new RCT is added to the network and a predetermined number of interactions with existing trials are imposed. The probability that an existing trial, *i*, receives a tie depends on its success during the previous testing in RCTs and is expressed in terms of survival hazard ratio (HR) and statistical significance:
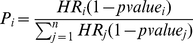
(1)where the summation is over all the nodes in the network at the current iteration, n.

#### Model 3: Random RCT interactions

The third and final model, assumes that trials interact at random. We construct five sets of Erdos-Renyi [21] random networks each of which is composed of 280 nodes representing each of the trials. The average number of ties in each set matches the average number of ties in the first 5 networks (4 constructed on the basis of shared characteristics and 1 on the basis of preferential attachment). The random networks are then compared with the shared characteristics and the preferential attachment networks.

### Network topology

To identify the topology of the RCT networks in order to compare the different structures, we computed the three most important measures of connectivity for each network: the average shortest path distance between all reachable nodes in a network, the global clustering coefficient, and the degree distribution. The shortest path distance shows how accessible the network is; small values are desirable for a tightly connected network. The global clustering coefficient measures the overall tendency of nodes to form clusters in which the connections of one node are themselves connected to each other forming distinct groups. The degree distribution is the distribution of the nodes' connections in the network. The pattern of the degree distribution is very important in network analysis since it shows the number of interactions each node (RCT) has. A glossary defining each of these terms is provided at the end of the manuscript.

### Node analyses

Not all the nodes in a network are of the same importance. Based on their position in the network, some nodes can interact more easily with other nodes, or are on many short paths between other pairs of nodes. These two properties are captured by the centrality measures of closeness and betweenness. Closeness measures the average distance a node has to all others in the network – shorter values mean greater ease of interaction with all others. Betweenness measures how important a node is in connecting other nodes [22] . Other centrality measures of importance are authority and hub [23]. Authority is a centrality measure that shows how influential a node is in the network while, a node is considered a hub if it is connected with many authorities. By computing centrality measures in the RCT networks we can identify the most central nodes and analyze their characteristics. A final node level measure is the local clustering coefficient. It measures the extent to which a node's connections are themselves connected to one another. High values mean the node is a member of a tightly knit cluster of nodes; low values, the opposite.

## Results

### Network topology

To identify the topology of the RCT networks we computed the average shortest path distance, the global clustering coefficient, and degree distribution for each network. We then compared these values to the corresponding values of a random network with the same number of nodes, and average number of ties.

Table 1 summarizes the values of global clustering coefficient and average shortest path distance for the networks studied and their corresponding random networks. The networks have been treated as undirected but within the constraint of time flow (i.e., only trials performed later in time could connect to trials conducted earlier in time). The preferential attachment network as well as the shared characteristics networks resulted in small average path distances, comparable to the distances in the corresponding random graphs but, global clustering coefficients much higher than their corresponding random networks (Table 1).

**Table 1:** Network topology characteristics for undirected networks.

Network	Average shortest path distance	Global Clustering coefficient	Number of nodes	Average number of ties
Group and Disease	1.80 ; 1.74*	0.68 ; 0.25*	279	71
Group and Treatment	1.57 ; 1.56*	0.73 ; 0.43*	279	120
Disease and Treatment	1.67 ; 1.61*	0.77 ; 0.38*	279	106
Group, Disease and Treatment	1.52 ; 1.52*	0.69 ; 0.47*	279	133
Preferential attachment	1.88 ; 1.87*	0.24 ; 0.13*	279	36

The asterisk corresponds to measures of random networks with the same number of nodes and ties.

**Figure 3:**
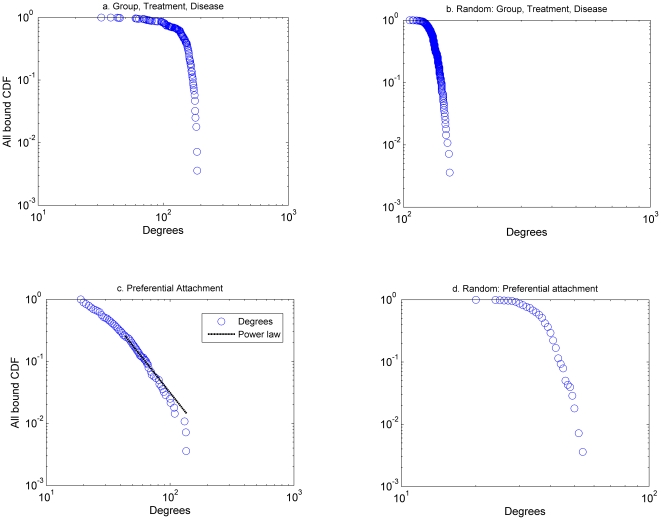
Connectivity distributions for different network configurations. The connectivity distribution for the shared characteristics network of group, treatment and disease (a) is described by a single scale distribution. The connectivity distribution for the preferential attachment network (c) is described by a power law distribution (The power law is of the form 
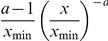
, with *α* = 2.83, *x_min_* = 27, and *p value* = 0.138). For brevity, we do not include the shared characteristics networks generated by the: group, treatment; group, disease; disease, treatment.

Such a pattern of connectivity corresponds to small world networks [24]. Characteristics of small world networks are: (a) small average shortest path distances, (b) large global clustering coefficients (larger than the corresponding random network), and (c) connectivity distributions described by either a scale free, broad scale or single scale distribution [25], [26]. The shared characteristics networks are small world networks with single scale connectivity distributions (figure 3a), while the preferential attachment network is a small world network with a power law (scale free) distribution of the form 
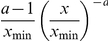
 (figure 3c). Since there is an uncertainty associated with the formation of the preferential attachment network we run 250 simulations out of which 225 have power law connectivity distribution with (*α*(*mean* = 2.8, *variance* = 0.18), *x_min_* (*mean* = 26, *variance* = 4.5) and *p value*>0.1 based on the algorithm presented in [10], which indicates that when *p value*>0.1, the power law is a plausible hypothesis for the data). The remaining 25 networks had *p value*<0.1.

Thus, the process of discovery of new therapeutics in cancer under the NCI umbrella, represented either as a shared characteristic network or a preferential attachment network, fits a pattern of connection that can be described as a small world network in which each trial is connected to any other trial in the network through just a few ties. This finding is probably not surprising since previous work showed that the structure of scientific collaboration networks often takes the form of small world networks [27], but it has never been studied in the setting of clinical research.

### Treatment discovery

**Figure 4:**
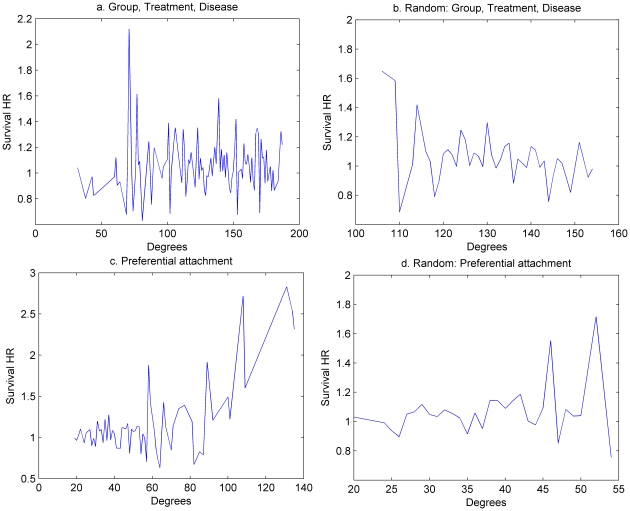
Average HR as a function of node connectivity for trials reporting survival outcomes. The plots (a, b, and d) show that there is no direct relationship between treatment success and connectivity. However, for the preferential attachment network (c), there is an increasing trend relationship between treatment success {as measured by survival HR (hazard ratio)} and connectivity arguing that better connected researchers may discover more life-saving treatments! For brevity, we do not include the networks generated by the shared characteristics: group, treatment; group, disease; disease, treatment.

We hypothesized that a positive relationship exists between the extent of a trial's interactions and treatment success. That is, the trials with many interactions will have greater treatment success than trials with few interactions. To test this hypothesis, we plotted the average value of survival hazard ratio for nodes as a function of their connectivity (degree). Figure 4a depicts the results for the shared characteristics network, while figure 4c shows the results for the preferential attachment network. Figures 4b,d illustrate the results for the corresponding random networks. Both figures 4a and 4c suggest that it is impossible to predict a particular trial's success based on its connectivity (degree). Furthermore, if RCT interactions are actually formed based on shared characteristics, then, despite the small world connectivity, the relationship between treatment success and RCT interactions is random with overall success rate slightly above 50% [8], [11]. Thus, trial success confined within the interactions at the level of the group, treatment, or disease is only slightly associated with the extent of connectivity. However, when trial success (HR) is plotted for the preferential attachment model (figure 4c), a different pattern emerges: the greater the extent of connectivity, the bigger is treatment success (HR) i.e. the higher are the chances that researchers discover new life-saving treatments! While this is a very intriguing result which can best explain the skewed distribution seen in figure 1, it could be argued that it is only reflection of the restrictions imposed on our model.

**Figure 5:**
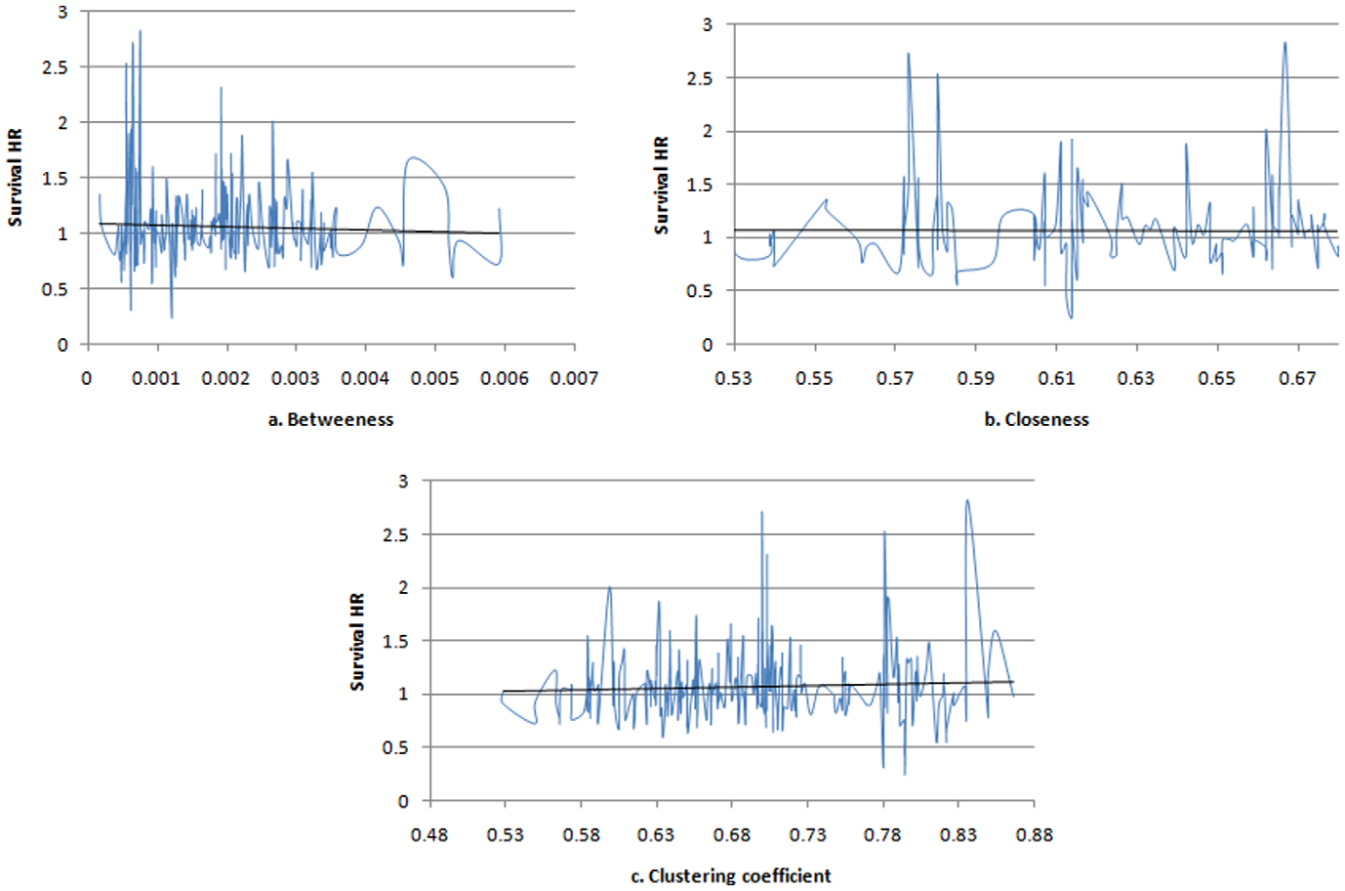
Average HR as a function of centrality measures for the shared characteristics network: group, disease, treatment. There is no identifiable pattern between survival HR and the various centrality measures.

**Figure 6:**
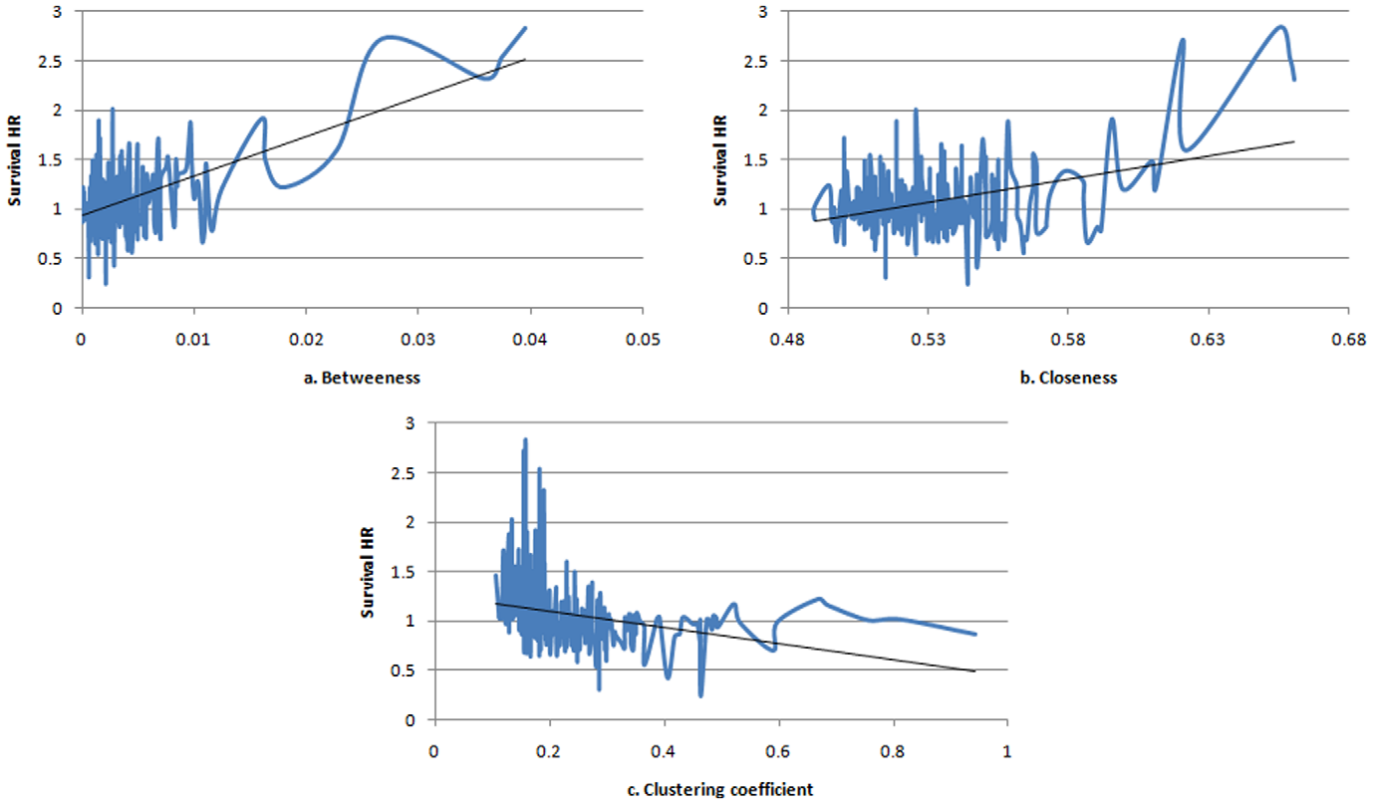
Average HR as a function of centrality measures for the preferential attachment network. There is an increasing trend between treatment success and the measures of betweenness and closeness (a, b), which implies that treatment success is not only a function of connectivity but also a function of node centrality and ease of access to relevant information. However, there is a decreasing trend between treatment success and local clustering coefficient (c). We speculated that nodes with high clustering coefficient are those which tend to interact within a closed group of trials (“silos” of information exchange) and consequently are the least successful.

To address the latter issue, we assess the relationships between treatment success and other centrality measures- the results that we believe could not be obviously predicted from the preferential attachment model. We, therefore, express treatment success as a function of closeness, betweenness, and local clustering coefficient. As expected, there is no identifiable pattern in the case of shared characteristics networks (figure 5). For the preferential attachment network, however, there is an increasing trend between treatment success and the measures of betweenness and closeness and a decreasing trend between treatment success and local clustering coefficient (figure 6). One interpretation of this finding is that those trials (researchers) with easy access to information (those with high betweenness and closeness) are more successful than others, while those researchers who tend to interact within a closed group (as so have high local clustering coefficients) are less exposed to good ideas/information and so are less successful [28].

### Node analyses

To identify characteristics of individual nodes in the network, we used the centrality measures described in the Methods section. Table 2 summarizes the characteristics for the nodes (trials) that present the highest centrality measures as well as the average survival HR values. We are interested in identifying the characteristics that make trial(s) distinctive.

**Table 2:** Characteristics of the most central trials.

Network	Measure	Group	Disease	Treatment	Av. HR
Group, Treatment, Disease	Betweenness Max = 0.006	ECOG	Leukemia	Definitive^*^	1.22
	Closeness Max = 0.75	ECOG	GI	Definitive^*^	1.22
	Authorities Max = 0.08	ECOG	GI(8/10); GYN(2/10)	Definitive^*^	0.97
	Hubs Max = 0.08	ECOG	GI(8/10); GYN(2/10)	Definitive^*^	0.97
Random network based on the Group, Treatment, Disease network	Betweenness Max = 0.002	RTOG	Lung	Definitive^*^	0.98
	Closeness Max = 0.69	ECOG	Lung	Definitive^*^	0.98
	Authorities Max = 0.06	RTOT (3/10); ECOG (5/10); GOG (1/10); SWOG (1/10)	GI(3/10); Lung (2/10); GYN (2/10); H&N Leukemia	Definitive^*^(6/10); Adjuvant (2/10); Supportive(1/10); Other (2/10)	0.96
	Hubs Max = 0.06	RTOT (3/10); ECOG (5/10); GOG (1/10); SWOG (1/10)	GI(3/10); Lung (2/10); GYN (2/10); H&N Leukemia	Definitive^*^(6/10)Adjuvant (2/10)Supportive(1/10)Other (2/10)	0.96
Preferential attachment	Betweenness Max = 0.04	RTOG	Prostate	Definitive^*^	2.82
	Closeness Max = 0.66	ECOG	Lung	Definitive^*^	2.31
	Authorities Max = 0.18	ECOG	Lung	Definitive^*^	2.31
	Hubs Max = 0.18	ECOG	Lung	Definitive^*^	2.31
Random network based on the Preferential attachment network	Betweenness Max = 0.068	SWOG	GI	Adjuvant	0.75
	Closeness Max = 0.554	SWOG	GI	Adjuvant	0.75
	Authorities Max = 0.089	ECOG (5/10); SWOG (2/10); NCCTG (1/10); CALGB (1/10); CHOG (1/10)	GI (3/10); CNS (2/10); Lung (2/10); Leukemia (1/10); Melanoma (1/10); Breast (1/10)	Definitive^*^(5/10); Adjuvant (3/10) Maintenance (1/10) Other	1.10
	Hubs Max = 0.089	ECOG (5/10); SWOG (2/10); NCCTG (1/10); CALGB (1/10); CHOG (1/10)	GI (3/10); CNS (2/10); Lung (2/10); Leukemia (1/10); Melanoma (1/10) Breast (1/10)	Definitive^*^(5/10) Adjuvant (3/10) Maintenance (1/10) Other	1.10

For brevity, we do not include the networks generated by the shared characteristics: group, treatment; group, disease; disease, treatment. The numbers in parentheses denote the memberships of each component in the group of nodes that present the same centrality measures.

It is interesting to note that, regardless of the approach used to form the RCT networks, the nodes with the highest centrality measures were the ones that studied curative/definitive treatments (Table 2) in solid tumors. This makes intuitive sense since large solid tumors (as opposed to hematological malignancies) can rarely be cured, and one would expect that trials attempting to test curative or more definitive treatments for these diseases would attract more attention from other investigators. Similarly, trials that were considered most central are the trials performed by ECOG (Eastern Cooperative Group) and studied curative/definitive type of treatments. This is probably not surprising since the ECOG is the largest NCI COG and is likely to have more influence on the trajectory of treatment discoveries than other NCI COGs. Likewise, curative/definitive treatments for solid tumors attracted more attention than more established therapies for lymphomas and other hematological malignancies. It should be noted that these treatments may not necessarily be less successful. Effective treatments for hematologic malignancies were discovered during early existence of the COG and hence it is not surprising that they received less attention during later decades of testing in RCTs conducted by the various COGs. This is particularly true as no major breakthroughs in the management of these diseases within the COG setting has occurred since the early 1970s.

## Discussion

One of the underlying premises of clinical research enterprise, including findings of new successful treatments, is that better scientific understanding should translate into improvement in patients' outcomes such as better survival. Such better scientific understanding is typically ensured via extensive social scientific networking that rely on interactions between past (e.g., via transfer of knowledge through scientific literature) and current researchers.

We argue that the interactions between researchers who conduct clinical trials are responsible for previously reported patterns in therapeutic discovery [8]. That is, treatment success in cancer is described by a power law distribution in which the majority of trials operate on the borderline of success and failure, while few trials are very successful [8].

Modeling interactions between researchers is a rather challenging process. We proposed three different approaches. First, we assume that RCT interactions are confined between trials in related fields such as cooperative group, type of disease or treatment (network with “shared characteristics”). Then, we generated RCT networks considering treatment success as the driving force of interactions. Finally, for comparison purposes, we assumed that RCTs interact at random.

Our results indicate that the networks created based on shared characteristics as well as those created based on treatment success are small world networks. Small worlds have been shown to describe other scientific collaboration networks [27]. However, this is the first time that they have been shown to apply to networks formed in clinical settings. The importance of the small world finding is that all trials are connected through a small number of ties enhancing the argument that treatment discovery is a social enterprise.

In addition, we show that, if RCTs are connected at random (figures 4d), or on a shared characteristic basis (figure 4a, 4b), dense interactions do not appear to translate into treatment success as measured in terms of improvement in cancer survival. On average, new treatments are only slightly superior to old ones: a finding explained by the equipoise hypothesis, which suggests that the requirement for uncertainty in clinical trials is what drives the RCT system, but which also predicts that new treatments are not very likely to be much more successful than the established ones [11], [29], [30]. However, the equipoise hypothesis does not explain the existence of a comparatively greater proportion of the small number of very successful trials among newly developed treatments [8].

A different picture emerges for the preferential attachment network: if trials are connected on an individual treatment success basis (figure 4c), then while for the majority of the trials the relationship between treatment success and connectivity seems random and governed by equipoise (figure 4c for degrees less than 100), there are few trials for which there is a proportional relationship between connectivity and success rate (figure 4c for degrees greater than 100). This finding agrees with our previously reported results that showed that treatment success in cancer is distributed as a power law function with the majority of the trials operating on the borderline between success and failure, and a small number of very successful trials [8]. The preferential attachment model provides an underlying mechanism that could explain this overall pattern of therapeutic discovery.

We believe that the mechanism responsible for the reported pattern of treatment discovery in cancer relates to the social interactions between RCTs as it stems from researcher's tendency to interact selectively with established and successful peers. It should be noted that the social interactions do not violate the equipoise requirement, rather they complement it. The findings indicate that the overall cancer RCT system maintains equipoise via unpredictability in the results at any individual trial, while providing the avenue for the researchers to increase their odds to discover new successful treatments which will go beyond 50∶50 odds predicted by the original equipoise hypothesis. We, therefore, argue that the social network analyses along with ethical analyses of equipoise presented in this paper provide further understanding of the principles that drive the treatment discovery process.

Our research has some limitations. The main limitation is that we have used interactions between RCTs as a proxy of the actual interactions between COG researchers. We had no way to identify the multitude of factors that actually influence researchers to determine why and what exactly they choose to study. Nevertheless, in the final analysis, many of these formal and informal mechanisms of interactions do converge to the factors we used in the analysis presented here. Second, we studied the process of social interactions within the closed system of NCI COGs. In reality, the NCI COG researchers interact with the outside biomedical research community and that may influence the types of research the NCI COG performs. However, the NCI COG is a very influential organization, and while it is probably influenced by outside factors, to some extent, it has its own platform for research development that, we believe, is accurately reflected in our analysis. To address the issue of the impact on non-NCI sources on the type of research performed by the NCI, we attempted to perform the citation analysis of RCT trials used in our analysis. Unfortunately, this proved unfruitful as the most publications did not cite the research leading to their proposals, thus making it impossible to create meaningful social interactions patterns.

We conclude that the treatment discovery process in RCTs could be explained by a small world network model according to which each trial is connected to any other trial in the network through a small number of steps. Furthermore, we present intriguing results that the richer the social interaction, as reflected in ease and importance of connections (closeness and betweenness), the greater the chance is that researchers may discover new life-saving treatments when connections are formed on the basis of preferential attachment. At the same time, trials which interact within “information silos” (as reflected by high local clustering coefficients), are associated with low survival HR arguing that the limited information exchange may be detrimental to the treatment discovery process!
